# Lossy and noisy channel simulation in computational ghost imaging by using noise-induced pattern

**DOI:** 10.1038/s41598-022-15783-6

**Published:** 2022-07-11

**Authors:** Jaesung Heo, Junghyun Kim, Taek Jeong, Sangkyung Lee, Yong Sup Ihn, Zaeill Kim, Yonggi Jo

**Affiliations:** Agency for Defense Development, Daejeon, 34186 South Korea

**Keywords:** Optics and photonics, Physics

## Abstract

We provide a method to evaluate effects of a lossy and noisy optical channel in computational ghost imaging (CGI) technique. Instead of preparing an external noise source, we simulate the optical channel with a basic CGI experiment using programmatically generated noise-induced patterns. By using our method, we show that CGI can reject a noise of which intensity is similar with an imaging signal intensity at a target. The results with our method are well matched with experimental ones including external noise source. This method would provide useful knowledge to analyze environmental effects in CGI without realization of the environment.

## Introduction

Ghost imaging (GI) is a novel imaging technique which exploits a correlation between two beams to obtain an image. Although its first demonstration was based on entanglement^1^, GI using classical light was demonstrated as well^2,3^, and theoretical studies revealed that it is not a quantum-originated phenomena^4–7^. Based on these studies, a computational GI (CGI) which makes use of only a single classical beam was proposed^8^. In CGI, a spatial light modulator (SLM) generates a spatial pattern-encoded beam instead of spatially correlated twin-beam. After the first proposal of CGI, there have been many extended studies such as compressive-sensing-enabled CGI^9–15^, three-dimensional imaging methods^16,17^, and light detection and ranging (LIDAR) systems based on CGI^18–22^.

In CGI, a spatial pattern encoded beam illuminates a target, and an intensity of a reflected beam from the target is measured. A target image can be constructed by averaging the signal light patterns weighted by the measured intensities. The outcome of CGI highly depends on spatial light patterns, and so far various studies to manipulate efficient patterns have been conducted^15,23–27^.

The robustness of GI against environmental effects, such as background noise^27–35^ and atmospheric turbulence^36–38^, has been theoretically studied and experimentally demonstrated. To observe the environmental effects, these demonstrations require large scale experimental settings such as an outdoor experiment, or an auxiliary implementation to generate artificial environmental effects. Here, we propose a method to simulate the environmental effects on CGI experiment without the use of auxiliary implementations. Because spatial patterns in CGI are generated and manipulated by software, it is possible to simulate an optical channel by programmatically including the channel effects into the patterns. In this paper, we investigate a simple optical channel, a lossy and noisy channel, where the noise energy is larger than the signal energy. This optical channel corresponds to an image-jamming attack against CGI system where strong thermal light illuminates the object or detector to disrupt the system. We compare the results of our method with the images obtained under the corresponding actual optical channel, and the validity of our method is discussed. This method would be exploited for simulating the effects of environment in CGI which is hard to be realized in a laboratory.

## Computational ghost imaging with noise-induced pattern

### Experimental setup

CGI exploits a beam, of which intensity is spatially modulated by using an SLM, and a single-pixel bucket detector. The beam illuminates a target, and the intensity of a transmitted or reflected beam is measured by the detector. After several repetitions with various spatial patterns and corresponding intensity outcomes, an image can be constructed based on a correlation *G* calculated as follows^8^:

1 $$\begin{aligned} \begin{aligned} G(\vec {x})=&\frac{1}{N}\sum _{n=1}^{N}I^{(n)} P^{(n)}(\vec {x})-\frac{1}{N}\sum _{n=1}^{N}I^{(n)}\times \frac{1}{N}\sum _{n=1}^{N}P^{(n)}(\vec {x}), \end{aligned} \end{aligned}$$

where *N* is the number of total trials, *I* is a measured intensity, *P* denotes a spatial pattern matrix, $\vec {x}$ is a position (*x*, *y*), and the superscript (*n*) denotes *n*-th trial. By subtracting the uncorrelated term, only the correlation between the intensity and pattern is left.

To enhance the image quality with a restricted number of shots, we exploit the Hadamard intensity patterns^9,15,39,40^. A $2^{2n}\times 2^{2n}$ Hadamard matrix is written in the following equation:

2 $$\begin{aligned} H_{2^{2n}}=H_{2^{2n-1}}\otimes H_{2}, \end{aligned}$$

where

3 $$\begin{aligned} H_{2}=\begin{bmatrix} 1 &{} 1 \\ 1 &{} -1 \end{bmatrix}, \end{aligned}$$

and $\otimes$ denotes tensor product. Hadamard patterns are obtained by reshaping each row of $H_{2^{2n}}$ into a $2^{n}\times 2^{n}$ square matrix. Because the intensity of light cannot be negative, we need two shots to represent one reshaped matrix^41^. In the first spatial pattern, the element value + 1 of the reshaped matrix corresponds to bright pixels of the Hadamard pattern and − 1 corresponds to dark pixels, while the second pattern is made by corresponding + 1 to dark pixels and − 1 to bright pixels.

**Figure 1:**
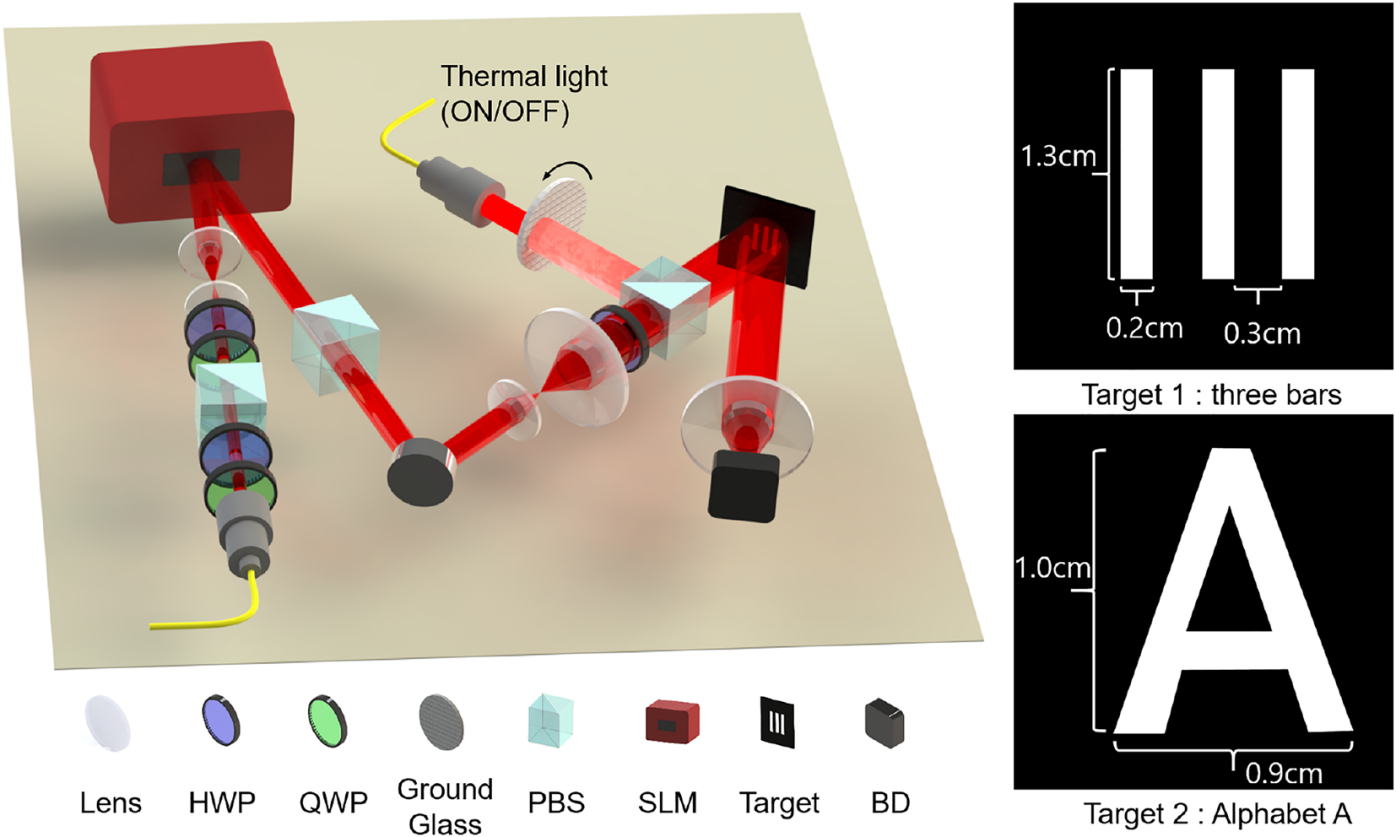
Experimental setup for CGI with two targets used for imaging, 3 bars and alphabet A. An H-polarized laser beam is reflected at the phase-controlling SLM. The phase-modulated beam becomes an intensity-modulated beam after the PBS. The modulated beam illuminates a target object and the intensity of received light from the target is measured by using a single-pixel bucket detector. For channel-simulated imaging, noise source is turned-off and noise-added patterns are displayed on the SLM. For the verification of our channel simulation, we turn on the thermal noise source consists of a laser and a rotating ground glass disk, and we conduct the imaging with the original Hadamard patterns. *HWP* half-wave plate, *QWP* quarter-wave plate, *PBS* polarizing beam splitter, *SLM* spatial light modulator, *BD* bucket detector.

Figure 1 shows a schematic diagram of our channel-simulated CGI experiment setup with two targets used for imaging, 3 bars and alphabet A. An 810 nm CW laser beam is horizontally polarized (H-polarized) by using a polarizing beam splitter (PBS), half and quarter wave plates (HWP and QWP), and it illuminates a phase-controlling SLM (Thorlabs, EXULUS-HD1). In our CGI experiment, $1024\times 1024$ SLM pixels are used to display $32 \times 32$ resolution Hadamard patterns, i.e., a unit area of the Hadamard patterns consists of $32\times 32$ SLM pixels. The Hadamard patterns are obtained from $H_{1024}$, and the total number of shots is therefore 2048. We place a PBS in front of the SLM to change the phase modulated beam into the intensity modulated beam. The SLM induces a $\lambda /2$ phase shift if the pattern is dark, which converts an H-polarized beam into V-polarized. The beam reflected at the bright part of patterns does not undergo any phase shift and remains H-polarized. Therefore, only a portion reflected from the bright part of the pattern passes through the PBS, resulting in an intensity-modulation. After the PBS, the modulated beam illuminates the target object, and the intensity of the reflected beam is measured by using a bucket photodiode. The channel simulation is conducted by displaying the noise-induced Hadamard patterns on the SLM.

A pseudo-thermal light source is exploited for the comparison which consists of an additional laser beam impinging on a rotating ground glass disk. The pseudo-thermal light is combined with the pattern encoded beam and illuminates the target.

### Lossy and noisy channel

**Figure 2:**
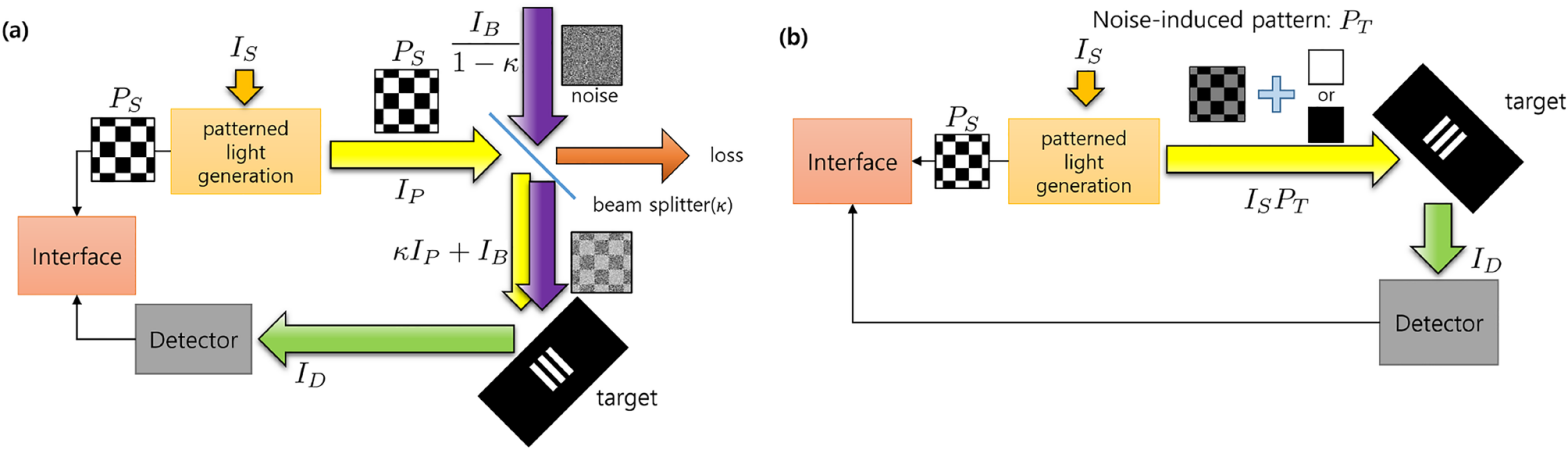
(**a**) A schematic diagram of CGI under a lossy and noisy channel. Starting from a light source with intensity $I_S$, the intensity of the initially patterned light is $I_P = \int _A \frac{I_S}{A} P_S(\vec x)d\vec x$, and that of environmental noise is $I_{B}/(1-\kappa )$. The two beams are mixed at a beam splitter whose reflectivity is $\kappa$, and the mixed beam illuminates the target. The reflected beam intensity is measured at the detector. (**b**) A schematic diagram of CGI with noise-induced patterns. The beam modulated by a noise-induced pattern ($P_T$) illuminates the target, while the original pattern ($P_S$) is used for the image calculation. $P_T$ is programmatically generated to have the same effects of noise and losses.

Figure 2a shows a schematic diagram of CGI under a lossy and noisy channel that we try to simulate. The intensity of the signal illuminating the target is $I_{S}$ when all the elements of $P(\vec {x})$ is one. For *n*-th pattern, the intensity becomes $I_P^{(n)}=\int _A \frac{I_S}{A} P^{(n)}(\vec x)d\vec x$, where *A* is the area of the pattern. Due to the lossy channel, the signal intensity decreases by a factor of $\kappa$, where $\kappa$ is the channel transmissivity. This can be modeled by using a beam splitter whose reflectivity is $\kappa$. At the beam splitter, environmental noise is mixed with the signal. To make constant noise of which average intensity is $I_{B}$ at the target, the intensity of noise before the beam splitter is set to $I_{B}/(1-\kappa )$. Then, the intensity of the combined light at the target becomes $\kappa I_{P}^{(n)}+I_{B}^{(n)}$, which implies that the light pattern has changed from the original Hadamard pattern due to the noise. Figure 2b shows our methodology, CGI with noise-induced patterns. We combine the effect of the loss and noise into the spatial pattern by a program, so the spatial profile of beam illuminating target has a dimmed Hadamard pattern with noise. For the image calculation in Eq. (1), the original Hadamard pattern ${P^{(n)}}$ is exploited.

The correlation for the image calculation is written in the following equation^8^:

4 $$\begin{aligned} \begin{aligned} G_{D}(\vec {x})=&\frac{1}{N}\sum _{n=1}^{N}I_{D}^{(n)} P^{(n)}_{S}(\vec {x})-\frac{1}{N}\sum _{n=1}^{N}I_{D}^{(n)}\times \frac{1}{N}\sum _{n=1}^{N}P^{(n)}_{S}(\vec {x})\\ =&\frac{1}{N}\sum _{n=1}^{N}\frac{1}{A}\int _{A} r(\vec {x'})\left[ \kappa I_{S} P^{(n)}_{S}(\vec {x'})+I_{B}^{(n)}\right] d\vec {x'}\,\,P^{(n)}_{S}(\vec {x})\\&-\frac{1}{N}\sum _{n=1}^{N}\frac{1}{A}\int _{A} r(\vec {x'})\left[ \kappa I_{S} P^{(n)}_{S}(\vec {x'})+I_{B}^{(n)}\right] d\vec {x'}\times \frac{1}{N}\sum _{n=1}^{N}P^{(n)}_{S}(\vec {x})\\ =&\frac{1}{N}\sum _{n=1}^{N}\int _{A} r(\vec {x'})\frac{\kappa I_{S}}{A} P^{(n)}_{S}(\vec {x'})d\vec {x'}\,P^{(n)}_{S}(\vec {x})-\frac{1}{N}\sum _{n=1}^{N}\int _{A} r(\vec {x'}) \frac{\kappa I_{S}}{A} P^{(n)}_{S}(\vec {x'})d\vec {x'} \times \frac{1}{N}\sum _{n=1}^{N}P^{(n)}_{S}(\vec {x})\\&+\frac{1}{N}\sum _{n=1}^{N}\int _{A} r(\vec {x'})\frac{I_{B}^{(n)}}{A}d\vec {x'}\, P^{(n)}_{S}(\vec {x})-\frac{1}{N}\sum _{n=1}^{N}\int _{A} r(\vec {x'})\frac{I_{B}^{(n)}}{A}d\vec {x'} \times \frac{1}{N}\sum _{n=1}^{N}P^{(n)}_{S}(\vec {x})\\ =&\kappa G_{S}(\vec {x})+G_{B}(\vec {x}), \end{aligned} \end{aligned}$$

where the subscripts *D*, *S*, and *B* denote the detected light, signal, and background noise, respectively, and $r(\vec {x})$ is the reflectivity of the target object. For simplicity, we assume that there is no loss except the lossy channel of which transmissivity is $\kappa$. $G_{S}(\vec {x})$($G_{B}(\vec {x})$) denote the correlations when there exists only signal(noise). If $\kappa =1$ and $I_{B}^{(n)}={\bar{I}}_{B}$ for all *n*, i.e., there is no loss and noise is constant without variance, $G_{D}(\vec {x})=G_{S}(\vec {x})$ satisfies, and therefore, the ideal computational ghost imaging is performed.

Let us analyze the case that $\kappa I_{P}^{(n)} < {\bar{I}}_{B}$ i.e., the signal intensity at the target is weak compared to noise. This corresponds to the case that there is an enemy who tries to interrupt our imaging by illuminating the target with strong jamming light. For maximum disruption of the imaging system, i.e., in order to maximize intensity fluctuation in the light detection, the jamming light is chosen as thermal light. An intensity distribution of thermal light is shown in the following equation^42^:

5 $$\begin{aligned} p(i)=\frac{1}{{\bar{I}}_{B}}e^{-i / {\bar{I}}_{B}}. \end{aligned}$$

This intensity distribution is super-Poissonian, so the intensity variance of the thermal light is larger than ${\bar{I}}_{B}$. If ${\bar{I}}_{B}$ is sufficiently large, the variance of the noise intensity can be comparable or larger than the differences in $\int _A \kappa I_{S} P_{S}^{(n)}(\vec {x})d\vec {x}/A$ for different *n*, and then, $G_{B}(\vec {x})$ is no longer negligible compared to $\kappa G_{S}(\vec {x})$^34,35^.

### Noise-induced patterns

In experiment, $I_{D}$ is determined by averaging *M* samples of intensity detection. That is, for each *n*-th pattern, light intensity is measured for *M* times, $I_{D}^{(n)(1)}, I_{D}^{(n)(2)},\dots , I_{D}^{(n)(M)}$, and we take $I_{D}^{(n)} = \frac{1}{M}\sum _{m=1}^{M} I_{D}^{(n)(m)}$. To analyze the effect of noise on $I_D$ only, consider the signal is off and $I_{D}^{(n)}$ is only dependent of noise, i.e., $I_{D}^{(n)} = I_{B}^{(n)} = \sum _{m=1}^{M}\frac{1}{M}I_B^{(n)(m)}$. Since each sample is recorded from light emerged by an identical source, statistical characteristics of *M* samples are identical. If sampling period is greater than the coherence time of light, we can assume *M* samples as independent and identically distributed random variables. Denoting this random variable as $I_B'$, mean and variance of each sample follow

6 $$\begin{aligned} E[I_B^{(n)(m)}] = E[I_B']\text { and }\text {var}[I_B^{(n)(m)}] = \text {var}[I_B'], \end{aligned}$$

for $m = 1,2,\dots ,M$, and thus,

7 $$\begin{aligned} E[I_B^{(n)}] = E[I_B'] \text { and }\text {var}[I_B^{(n)}] = \frac{1}{M} \text {var}[I_B'], \end{aligned}$$

where var(*X*) denotes a variance of *X*. For light modulated by pattern to imitate this noise behavior, mean and variance of such light detected must be equal to Eq. (7).

To simulate this noise behavior, we introduce noise-induced patterns $P_{T}(\vec {x})$, which consist of Hadamard patterns $P_S$ with a loss rate $\kappa$ and noise-imitating patterns(NIP) $P_B$, i.e., $P^{(n)}_{T}(\vec {x})=\kappa P^{(n)}_{S}(\vec {x})+P^{(n)}_{B}$ for *n*-th Hadamard pattern. Then, the correlation is calculated as follows:

8 $$\begin{aligned} \begin{aligned} G_{D}(\vec {x})=&\frac{1}{N}\sum _{n=1}^{N}I_{D}^{(n)}P^{(n)}_{S}(\vec {x})-\frac{1}{N}\sum _{n=1}^{N}I_{D}^{(n)}\times \frac{1}{N}\sum _{n=1}^{N}P^{(n)}_{S}(\vec {x})\\ =&\frac{1}{N}\sum _{n=1}^{N}\int _{A}r(\vec {x'})\frac{I_{S}}{A}P^{(n)}_{T}(\vec {x'})d\vec {x'}\, P^{(n)}_{S}(\vec {x})-\frac{1}{N}\sum _{n=1}^{N}\int _{A}r(\vec {x'})\frac{I_{S}}{A}P^{(n)}_{T}(\vec {x'})d\vec {x'}\times \frac{1}{N}\sum _{n=1}^{N}P^{(n)}_{S}(\vec {x})\\ =&\frac{1}{N}\sum _{n=1}^{N}\int _{A}r(\vec {x'})\frac{I_{S}}{A}\left[ \kappa P^{(n)}_{S}(\vec {x'})+{P_{B}^{(n)}}\right] d\vec {x'}\, P^{(n)}_{S}(\vec {x})\\&-\frac{1}{N}\sum _{n=1}^{N}\int _{A}r(\vec {x'})\frac{I_{S}}{A}\left[ \kappa P^{(n)}_{S}(\vec {x'})+{P_{B}^{(n)}}\right] d\vec {x'}\times \frac{1}{N}\sum _{n=1}^{N}P^{(n)}_{S}(\vec {x})\\ =&\frac{1}{N}\sum _{n=1}^{N}\int _{A}r(\vec {x'})\frac{I_{S}}{A}\kappa P^{(n)}_{S}(\vec {x'})d\vec {x'}\, P^{(n)}_{S}(\vec {x})-\frac{1}{N}\sum _{n=1}^{N}\int _{A}r(\vec {x'})\frac{I_{S}}{A}\kappa P^{(n)}_{S}(\vec {x'})d\vec {x'}\times \frac{1}{N}\sum _{n=1}^{N}P^{(n)}_{S}(\vec {x})\\&+\frac{1}{N}\sum _{n=1}^{N}\int _{A}r(\vec {x'}){\frac{I_{S}}{A}P_{B}^{(n)}}d\vec {x'} P^{(n)}_{S}(\vec {x})-\frac{1}{N}\sum _{n=1}^{N}\int _{A}r(\vec {x'}){\frac{I_{S}}{A}P_{B}^{(n)}}d\vec {x'}\times \frac{1}{N}\sum _{n=1}^{N}P^{(n)}_{S}(\vec {x}). \end{aligned} \end{aligned}$$

Then, Eqs. (4) and  (8) are the same if the following condition is true:

9 $$\begin{aligned} \int _{A}r(\vec {x})I_{B}^{(n)}d\vec {x}= \int _{A}r(\vec {x})I_{S}P_{B}^{(n)}d\vec {x}. \end{aligned}$$

Thus, $I_S P_{B}$ is necessary to have the same average and variance with those of $I_{B}$ under Eq. (7).

To analyze the effect of NIP only, let us consider the case $P^{(n)}_{T}(\vec {x})=P^{(n)}_{B}$ and assume $r(\vec {x}) = 1$ for all $\vec {x}$ for simplicity. Suppose that the detected intensity of *n*-th NIP-modulated light, $I_D^{(n)} = I_S P_B^{(n)}$, is given by averaging $M'$ intensity samples, i.e., $I_S P_B^{(n)} = \sum _{m'=1}^{M'} \frac{1}{M'} I_S P_B^{(n)(m')}$. Since the source light intensity $I_S$ is assumed to be constant, statistics of NIP-modulated light is determined by NIP only. With random variable that follows the statistics of NIP as $P_B'$, statistics of independent and identically distributed $M'$ samples can be represented by the following:

10 $$\begin{aligned} \begin{aligned} E\left[ I_S P_B^{(n)(m')}\right]&= I_S\cdot E\left[ P_B^{(n)(m')}\right] = I_S E\left[ P_B'\right] ,\\ \text {var}\left[ I_S P_B^{(n)(m')}\right]&= {I_S}^2 \text {var}\left[ P_B^{(n)(m')}\right] = {I_S}^2 \text {var}\left[ P_B'\right] . \end{aligned} \end{aligned}$$

Thus, statistics of detected intensity becomes

11 $$\begin{aligned} \begin{aligned} E\left[ I_S P_B^{(n)}\right]&= I_S E\left[ P_B'\right] ,\\ \text {var}\left[ I_S P_B^{(n)}\right]&= \frac{1}{M'} {I_S}^2 \text {var}\left[ P_B'\right] . \end{aligned} \end{aligned}$$

If we choose the statistics of NIP that gives var$[P_B']=\left( E[P_B']\right) ^2$, then we can obtain the following conditions from Eqs. (7) and  (11):

12 $$\begin{aligned} \begin{aligned} E\left[ I_B^{(n)}\right]&=E\left[ I_SP_B^{(n)}\right] = I_S E\left[ P_B'\right] , \\ \text {var}\left[ I_B^{(n)}\right]&= \text {var}\left[ I_SP_B^{(n)}\right] = \frac{1}{M'} \left( {I_S} E\left[ P_B'\right] \right) ^2, \end{aligned} \end{aligned}$$

so

13 $$\begin{aligned} M' = \frac{\left( E\left[ I_B^{(n)}\right] \right) ^2}{\text {var}\left[ I_B^{(n)}\right] }. \end{aligned}$$

The first condition in Eq. (12) implies matching of mean intensity, and the second condition in Eq. (12) gives the value of $M'$ in terms of measurable quantities of jamming noise. This implies that any noise can be simulated by NIP with given statistics.

Such NIP statistics can be realized by the combination of two binary patterns, fully bright or fully dark pattern satisfying $I_S P_{\text {bright}} = I_S$ or $I_S P_{\text {dark}} = 0$, respectively. For each $M'$ samplings, $P_{\text {bright}}$ or $P_{\text {dark}}$ are randomly chosen with probability 0.5, which results in Bernoulli distribution. Mean and variance of NIP are following:

14 $$\begin{aligned} \begin{aligned} E\left[ P_B^{(n)(m')}\right]&=\frac{1}{2} (P_{\text {bright}} + P_{\text {dark}}) = \frac{1}{2},\\ \text {var}\left[ P_B^{(n)(m')}\right]&= \frac{1}{4} = \left( E\left[ P_B^{(n)(m')}\right] \right) ^2, \end{aligned} \end{aligned}$$

for $$m' = 1,2,\ldots ,M'$$.

To generate $P_B^{(n)}$ programmatically, effective $P_B^{(n)}$ which is equivalent to averaging the results of $M'$ NIP samples is required. Let the weighting constants determined by the sum of $M'$ Bernoulli processes with probability 0.5 be $C = B(M',0.5)$. Then, $P_B^{(n)}$ is calculated as:

15 $$\begin{aligned} P_B^{(n)}=\frac{C}{M'}P_{\text {bright}}+\frac{M'-C}{M'}P_{\text {dark}}. \end{aligned}$$

With NIP to imaging patterns $P_S$, any lossy and noisy channel can be simulated with proper $M'$ combinations of the two binary patterns.

In our experiment, thermal light is used for jamming. That is, $I_B'$ follows the Bose–Einstein distribution described by Eq. (5). In this case, var$[I_B'] = E[I_B']\left( E[I_B']+1\right) \approx \left( E[I_B']\right) ^2$, where the last approximation is valid when $E[I_B']\gg 1$, which is the case of classical light. Then, Eq. (12) becomes as follows:

16 $$\begin{aligned} \begin{aligned} E[I_B^{(n)}]&= E[I_B'] = I_S E[P_B'],\\ \text {var}[I_B^{(n)}]&= \frac{1}{M}\left( E[I_B']\right) ^2 = \frac{1}{M'} \left( I_S E[P_B']\right) ^2 , \end{aligned} \end{aligned}$$

and these two conditions give $M=M'$.

### Noise-induced pattern for channel simulation

In this section, we consider how to realize noised-induced patterns $P_T$ experimentally. Intuitively, it is expected that combination of the original Hadamard patterns and spatially speckled patterns of thermal light is enough to construct noise-induced patterns. However, an SLM consists of a limited number of pixels, and it can display only a discrete intensity which can be described with an unsigned 8-bit integer. Due to these imperfection, a spatial pattern of thermal light cannot be perfectly mimicked with an SLM. If we perform the combination, we can check that measured intensity in the photodiode cannot follows a thermal light distribution. Therefore, we need another method to simulate a lossy and noisy optical channel. By displaying noise-induced patterns $P^{(n)}_{T}(\vec {x})$ on the SLM, we can simulate the imaging under lossy and noisy channels. However, the SLM cannot display a pattern that has elements exceeding the maximum available pixel value. Therefore, the noise-induced pattern should be normalized to $P^{(n)}_{T}(\vec {x})/I_{N}$, where $I_{N}$ is a normalization factor determined by the pixel value limit given by the SLM. To compensate this, the incident beam intensity should be $I_{N}I_{S}$ rather than the original intensity $I_{S}$.

The range of pixel values of our SLM(Thorlabs, EXULUS-HD1) is from 0 to 255. However, our SLM was unstable displaying pixel value which is not binary(0 or 255). We thus separately display the Hadamard patterns and NIP so that both have pixel values either 0 or 255: $P_{\text {bright}}$ and $P_{\text {dark}}$ for NIP, and $P_S$ for Hadamard patterns. For our purpose, we reformulate $P_T I_S$ in terms of $P_S I_{SS} + P_{\text {bright}} I_{S,\text {bright}} + P_{\text {dark}} I_{S,\text {dark}}$ as follows:

17 $$\begin{aligned} \begin{aligned} \frac{P_T^{(n)}(\vec x)}{I_N}\cdot (I_N I_S)=&\kappa P_S^{(n)}(\vec x)I_S+ P_B^{(n)}I_S\\ =&P_S^{(n)}(\vec x) \cdot \kappa I_S + P_{\text {bright}} \cdot \frac{C}{M'}I_S + P_{\text {dark}} \cdot \frac{M'-C}{M'}I_S. \end{aligned} \end{aligned}$$

Then, we can obtain the following conditions:

18 $$\begin{aligned} I_{SS}=\kappa I_S\text {, } I_{S,\text {bright}}=\frac{C}{M'}I_S\text {, and } I_{S,\text {dark}}=\frac{M'-C}{M'}I_S. \end{aligned}$$

The experimental step for this separation is as follows: first, we display $P_S$ on the SLM and reduce the intensity by a factor of $\kappa$ by HWP and PBS, measuring the intensity of $I_{SS}$. Next, we display $P_{\text {bright}}$ on the SLM and sample the intensity for *C* times without applying $\kappa$. After summing up the measured intensities and dividing it by $M'$ results in $I_{S, \text {bright}}$, the same step can be performed to obtain $I_{S,\text {dark}}$ by displaying $P_{\text {dark}}$ with $M'-C$ times sampling. By this method, imaging under a lossy and noisy channel can be simulated using only binary pixel values, making not only SLM but also digital micromirror device (DMD) to be able to perform this simulation.

## Results

**Figure 3:**
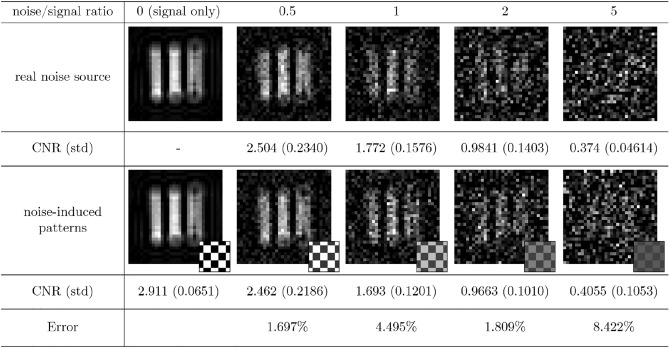
Three bar images obtained by using CGI with an external thermal noise source and those of CGI with noise-induced patterns. The small pictures show examples of the noise-induced patterns. We calculate contrast-to-noise ratio (CNR) and its standard deviation (std).

**Figure 4:**
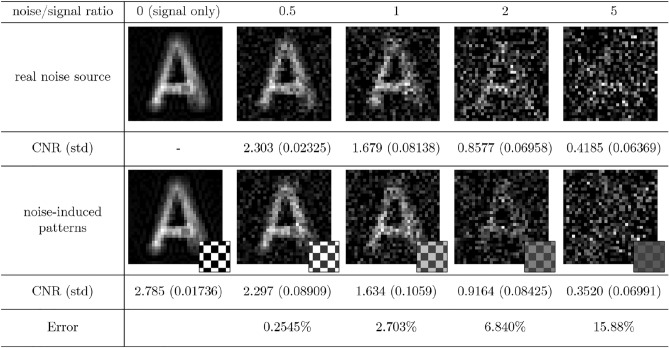
Alphabet A images obtained by using CGI with an external thermal noise source and those of CGI with noise-induced patterns. The small pictures show examples of the noise-induced patterns. We calculate contrast-to-noise ratio (CNR) and its standard deviation (std).

To quantify a quality of result images, we exploit contrast-to-noise ratio (CNR) shown in Eq. (19):

19 $$\begin{aligned} \text {CNR}=\frac{|\mu _{1}-\mu _{0}|}{\sqrt{(\sigma _{1}^{2}+\sigma _{0}^{2})/2}}, \end{aligned}$$

where $\mu _{j}$ and $\sigma _{j}$ denote mean value and standard deviation of image pixel values, respectively, and they are calculated for the target image ($j=1$) and for the background noise ($j=0$)^43^.

Figures 3 and  4 show the results of CGI under the real thermal noise injection and those with noise-induced patterns. The noise-induced patterns are also shown with the simulated images. Ratios of the signal and noise intensity $I_{B}/\kappa I_{S}$ ranging from 0 to 5 are exploited to compare the two methods. For each condition, imaging is performed for 4 times, and mean and standard deviation of CNRs of each condition are given below the corresponding images. Figure 3 is the result of imaging three bars. The mean and variance of detected real thermal noise are 0.2151 V and $2.610\times 10^{-4}$, respectively, giving $M'=177$ according to Eq. (13). With this sampling number, NIP-modulated light gives 0.2146 V for mean and $2.586\times 10^{-4}$ for variance. Figure 4 is the result with object alphabet *A*. The mean and variance of real noise are 0.2150 V and $2.563\times 10^{-4}$, respectively, giving $M'=180$. The mean of NIP-modulated light is 0.2149 V and the variance is $2.412\times 10^{-4}$.

The CNRs of CGI with noise-induced patterns and real noisy channel imaging agree well with error less than 7% when the noise-to-signal intensity ratio is less than 5, but error increases to maximum 16% when the ratio is 5. As the noise increases, randomness affects large part of imaging. To converge CNR with such randomness, imaging for 4 times is not enough. This results in huge error for ratio 5. Overall, our method predicts the actual result image within $16\%$ error.

## Conclusion and discussion

In this paper, we proposed a method to simulate an optical channel in CGI. To predict the degradation arose from a lossy and noisy channel, we first calculated the effect of a thermal noise from its statistics and included them in spatially modulated patterns. We compared the image obtained by our method with the image obtained by the actual noise-embedded imaging. The comparison of two result images under various noise conditions showed that our method can predict the effects of noisy channel within a $7\%$ error when the noise is up to 2 times brighter than the signal. We expect this method would be extended to an optical channel simulation of CGI when the environments are hard to be implemented in a laboratory such as atmospheric turbulence channel^36–38^.

An accuracy of our method can be improved with an advanced SLM. For example, there are commercial SLMs that can display a 10-bit grayscaled image such as SENTEC SLM-200, and there was the research to display 12-bit grayscaled image on Hamamatsu X10468-01^44^. By increasing SLM grayscale levels, an approximation of the thermal noise becomes more accurate, and therefore, we expect that simulation results should be closer to the realistic ones.

One may wonder what the difference is between our method and a fully-programmed CGI simulation without any experiment. For the fully-programmed simulation, it is necessary to include experimental parameters of the devices in the program such as a photodetection efficiency. Unlike this approach, in our method, characteristics of devices are naturally included in a result image, and therefore, an analysis of device parameters is not necessary except the SLM.

## Data Availability

The datasets generated and/or analyzed during the current study are not publicly available due to the security policy of the Ministry of National Defense of South Korea but are available from the corresponding author on reasonable request.
